# The complete mitochondrial genome sequence of *Alcippe ruficapilla* (Passeriformes: Timaliidae) and phylogenetic position

**DOI:** 10.1080/23802359.2019.1704646

**Published:** 2020-01-09

**Authors:** Qingqing Chong, Yanyan Chen, Juntao Liao, Heng Xiao, Shanyuan Chen

**Affiliations:** a School of Life Sciences, Yunnan University, Kunming, China;; b National Demonstration Center for Experimental Life Sciences Education, Yunnan University, Kunming, China

**Keywords:** *Alcippe ruficapilla*, mitogenome sequence, next-generation sequencing

## Abstract

In this work, we first reported the complete mitochondrial genome (mitogenome) sequence of *Alcippe ruficapilla* by next-generation sequencing (NGS). The complete mitogenome of *A. ruficapilla* was 16,941 bp in length and contained the typical structure of 13 protein-coding genes, 2 ribosomal RNA genes, 22 transfer RNA genes (tRNAs) and 2 no-coding control regions (D-loop). Nucleotide composition of the whole mitogenome was 29.2% A, 24.2% T, 31.87% C, and 14.8% G, respectively. Phylogenetic analysis indicated that *Alcippe ruficapilla* gathered into a single branch with *Montifrigilla henrici* and *Acrocephalus scirpaceus* with strong support.

Spectacled Fulvetta (*Alcippe ruficapilla*) is a kind of endemic bird with important economic and scientific research value. Its taxonomic status has been updated as *Fulvetta ruficapilla* in Birdlife checklist version 09 (December 2016). This bird species only distributes in northwest and southwest China and Laos. In this work, we first reported the complete mitochondrial genome (mitogenome) sequence of *A. ruficapilla,* which would contribute to enrich the conservation genetics resource and promote its related research.

We collected the sample in Yunnan Provence, southwestern China (25.04°N, 102.73°E). The entire specimen was stored in freezer at –40 °C and registered in the Zoological Specimen Museum of Yunnan University under the voucher number YNUBD2016AF01. Being similar to previous studies (Chen et al. 2018; Li et al. 2018), genomic DNA was extracted from muscle tissue by Dneasy Blood and Tissue Kit (QiaGen) and DNA shotgun library was constructed and sequenced with Illumina Miseq platform (Illumina, USA). Subsequently, the mitogenome of *A. ruficapilla* was assembled with Trinity v2.3.2 (Haas et al. 2013) and SPAdes (Bankevich et al. 2012). All 13 protein coding genes and 2 ribosomal RNA genes were identified using DOGMA (Wyman et al. 2004). The 22 transfer RNA genes were identified by tRNAscan-SE (Lowe and Eddy 1997). We employed an online molecular biology tool CGVIEW to draw the mtDNA genome map. The complete mitogenome of *A. ruficapilla* had been deposited in GenBank under accession number MK988446.

The complete mitogenome was 16,941 bp in length, with the overall base composition of 29.2% A, 24.2% T, 31.8% C, and 14.8% G. It contains 13 protein-coding genes (PCGs), 2 ribosomal RNA genes (rRNA), 22 transfer RNA genes (tRNA), and 2 control regions (D-loop). Except for NADH6 gene and 8 tRNA genes (tRNA-Gln, Trna-Ala, tRNA-Asn, tRNA-Cys, Trna-Try, tRNA-Ser, tRNA-Pro, and tRNA-Glu), all others genes were located on heavy strand (H-strand). In these genes, except COI started with GTG, others genes started with ATG. In addition, these genes had four types of termination codons, including TAA for NADH2, COII, ATP8, ATP6, NADH3, NADH4L, NADH5 and CYTB; AGG for COI; AGA for NADH1, TAG for NADH6; and incomplete stop codon (T–) for COIII and NADH4.

To investigate the phylogenetic position of *A. ruficapilla*, phylogenetic tree was reconstructed based on the concatenated sequences of 13 protein coding genes (PCGs) from 12 Passeroforme species using maxmium likelihood (ML) method implemented in MEGA 6.0. As shown in the phylogenetic tree (Figure 1), the result showed that *A. ruficapilla* gathered into a single branch with *Montifrigilla henrici* and *Acrocephalus scirpaceu*s with strong support, being consistent with that of Alström et al. (2006). Our data would provide useful information for application in conservation genetics for the species.

**Figure 1.**
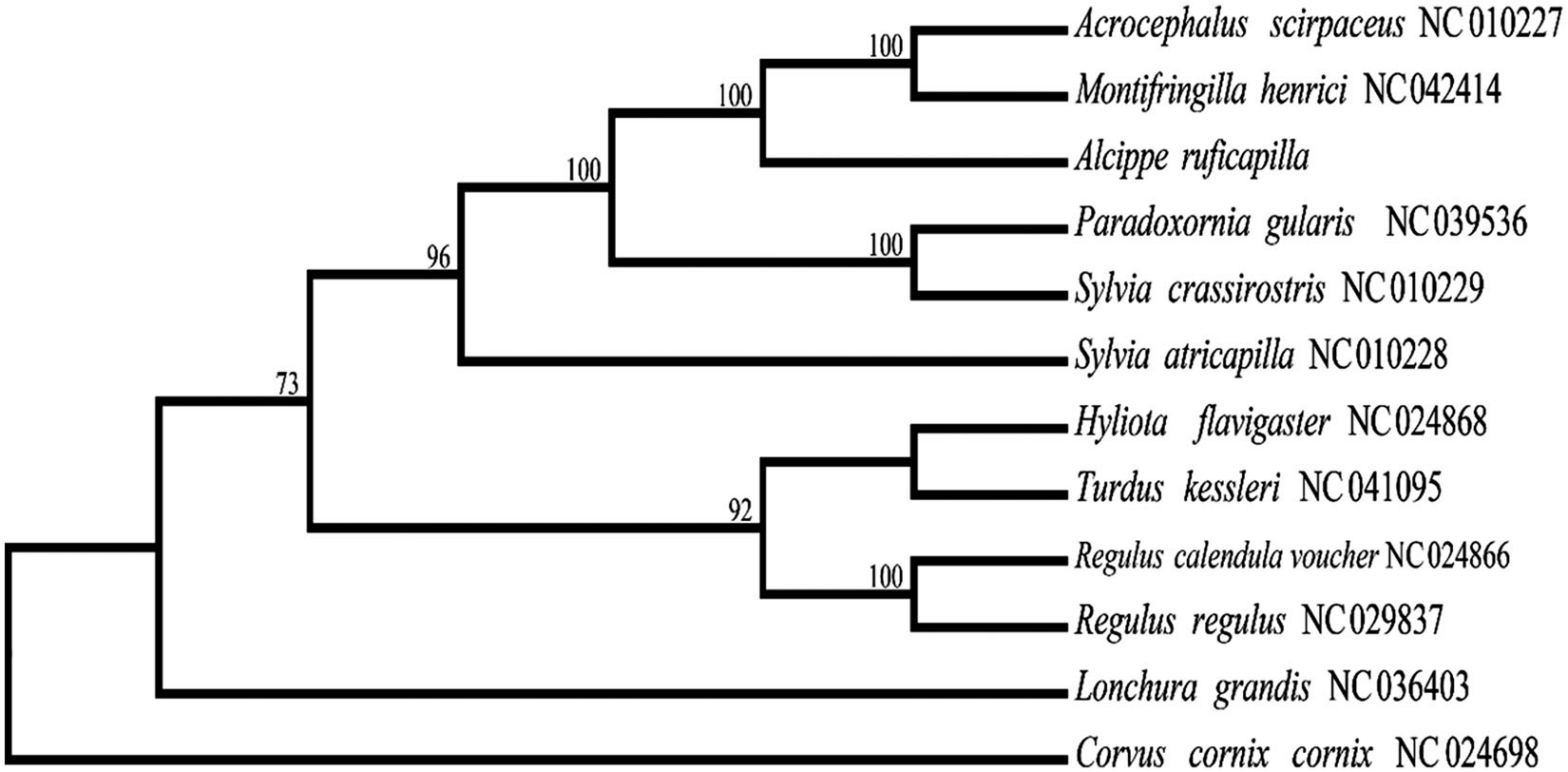
The phylogenetic relationship among 12 Passeriforme species based on the 13 concatenated sequences of mitochondrial PCGs using maximum likelihood method. The number after the species name is GenBank accession number. Numbers in the nodes represent support values with 1000 bootstrapping replications.
